# Tryptophan metabolic pathway and neopterin in asthmatic children in clinical practice

**DOI:** 10.1186/s13052-019-0699-6

**Published:** 2019-08-28

**Authors:** Amelia Licari, Dietmar Fuchs, Gianluigi Marseglia, Giorgio Ciprandi

**Affiliations:** 1 Pediatrics Clinic, Department of Pediatrics, Ospedale San Matteo, University of Pavia, Pavia, Italy; 2 Biological Chemistry, Biocenter, Innsbruck Medical University, Innsbruck, Austria; 3 Allergy Clinic, Casa di Cura Villa Montallegro, Via Montezovetto 27, 16132 Genoa, Italy

**Keywords:** Tryptophan, Kynurenine, Neopterin, Asthma, Children

## Abstract

Tryptophan metabolic pathway is involved in pathogenic mechanisms of asthma. This study aimed to evaluate tryptophan metabolites and neopterin in a group of asthmatic children. Tryptophan metabolites and neopterin were measured in asthmatic children (121, 71 males, 50 females, mean age 11.6 + 3.2 years) and well-matched healthy controls (63, 32 males, 31 females, mean age 10.7 + 2.6 years). Tryptophan, kynurenine, and neopterin levels were significantly higher in asthmatic children than in healthy controls (*p* < 0.01; *p* < 0.01; *p* = 0.0015 respectively). Tryptophan metabolites and neopterin are increased in asthmatic children; these mediators underline the complex mechanisms involved in the immune response in asthma.

## Introduction

Allergic asthma is supported by type 2 inflammation and down-regulated type 1-response with reduced secretion of interferon-γ [1]. Type 2 inflammation is paradigmatically characterized by eosinophilic airway infiltration [2]. Actually, phenotyping and endotyping asthmatic patients is fundamental to tailor the most appropriated personalized therapy [3]. In this regard, it has been recently investigated the role of CD11b^+^Ly6G^+^ neutrophilic cells that were able to suppress airway inflammation in allergic mice [4]. So, the adoptive cellular transfer of suppressive neutrophilic cells may represent a possible way against allergic airway inflammation.

On the other hand, interferon-γ is a strong inducer of the enzyme indoleamine 2,3-dioxygenase (IDO) able to degrade the essential amino acid tryptophan as part of the antiproliferative strategy of immunocompetent cells, e.g. to halt the growth of infected and malignant cells [5].

Recently, it has been pointed out the IDO pathway as central to allergic inflammation [6]. In addition, higher serum tryptophan concentrations were described in patients with seasonal allergic rhinitis (SAR) compared to blood donors and higher baseline tryptophan concentrations were associated with poor response to specific immunotherapy [7]. Moreover, tryptophan concentrations were found to be higher in patients only off pollen season but not in season [8]. Notably, the kynurenine to tryptophan ratio (Kyn/Trp, and index of tryptophan breakdown) was unchanged, and tryptophan metabolism changes were independent from neopterin concentrations, a marker of type 1 immunity. Instead, serum neopterin concentrations were even slightly higher than in, e.g., populations of blood donors and healthy controls [9]. Accordingly, the increase of tryptophan levels seems to be independent from neopterin concentrations and thus type 1 immunity. Still subnormal IDO activity could be also involved in the increase of tryptophan levels, since also concentrations of serotonin, another substrate of IDO, increased in SAR patients and were strongly related with behavioural impairment, assessed by quality of life questionnaires [10].

Another intriguing aspect is the specific interaction of nitric oxide (NO) with IDO as fractional exhaled NO is a surrogate biomarker of type 2 inflammation [11]. Likewise, it has been demonstrated that NO slows down the expression and activity of the heme enzyme IDO [5]. At least our observations would be explainable when tryptophan concentrations increase due to suppression of IDO activity by NO. Notably, no inhibition of NO is known for GTP-cyclohydrolase I, the key enzyme for neopterin production. Thus, this background corresponds well with the independent development of tryptophan breakdown and neopterin concentrations in patients with allergic disorders.

Therefore, this scenario appears very complex, but could suggest next relevant consequences for future therapeutic strategies. In this regard, a crucial piece of this puzzle still lacks, such as the in-depth evaluation of IDO and neopterin pathway in childhood asthma. Indeed, a recent study investigated the concentrations of IDO metabolites (i.e. tryptophan and kynurenine) in 30 asthmatic children [12]. The expression of IDO was significantly lower in childhood allergic asthma, particularly in children with high FeNO levels, but there was no significant relationship between IDO levels and asthma severity. As these outcomes are conflicting with previous reports and did not evaluated neopterin secretion, the current study aimed to evaluate these mediators in a group of asthmatic children visited in a real-life setting.

## Materials and methods

The current study was designed as cross-sectional and was conducted in a real-world setting. The study included 121 consecutive children (71 males, 50 females, mean age 11.6 + 3.2 years) with allergic asthma and visited for the first time at a third-level paediatric clinic. They were compared with a well-matched group of 63 healthy children (32 males, 31 females, mean age 10.7 + 2.6 years). The procedure was approved by the Ethics Committee and parents signed an informed consent.

Inclusion criteria were: age between 6 and 14 years, both genders, asthma diagnosis. Exclusion criteria were: use of medications able to interfere with the interpretation of the results, current respiratory infections, severe disorders able to interfere with the interpretations of the results.

Asthma diagnosis was performed according to the Global Initiative for Asthma [13].

IDO pathway metabolites, such as tryptophan and kynurenine, and neopterin were measured as previously described in detail [7–10]. Tryptophan and kynurenine concentrations were measured by high-performance liquid chromatography (HPLC) using 3-nitro-Ltyrosine as internal standard. Tryptophan was detected by a fluorescence detector (ProStar, Model 360, Varian, Palo Alto, CA) at an excitation wavelength of 285 nm and an emission wavelength of 365 nm. A Shimadzu SPD-6A UV-detector (Shimadzu, Kyoto, Japan) in flow stream series connection was used for detection of both kynurenine and nitrotyrosine at a wavelength of 360 nm. To estimate IDO activity, kyn/trp was calculated (expressed as mol kynurenine per mmol tryptophan). Neopterin concentrations were determined by ELISA (BRAHMS, Hennigsdorf, Germany) according to the manufacturer’s instructions, with a detection limit of 2 nmol/l.

Data are reported as mean with + Standard Deviation. Difference in the mean values was evaluated with the Wilcoxon signed rank test. Statistica software 9.0 (StatSoft Corp., Tulsa, OK, USA) was used.

## Results

**Fig. 1:**
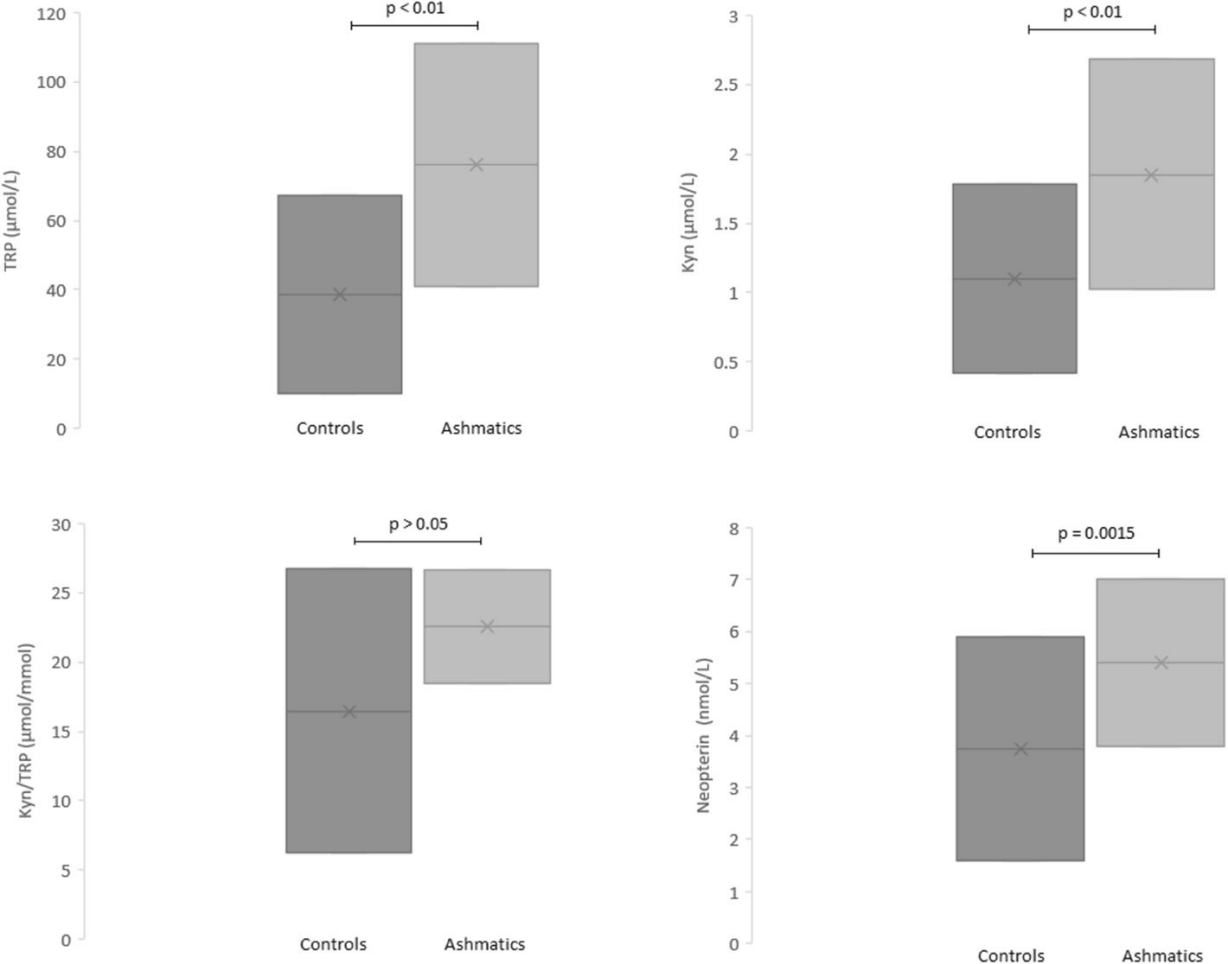
Serum concentration of tryptophan (upper left), kynurenine (upper right), kynurenine-to-tryptophan ratio (kyn/trp) (lower left), and neopterin lower right) in patients with seasonal allergic rhinitis evaluated during or outside of the pollen season and in healthy subjects. Data are represented as medians (horizontal lines), interquartile ranges (boxes), and ranges (vertical lines), excluding outliers (*p* values, Kruskal–Wallis test, and p values of individual group comparisons are given)

Figure 1 and Table 1 show that asthmatic children had higher tryptophan and kynurenine values together neopterin than healthy children, whereas the ratio tryptophan/kynurenin was similar in the two groups. Notably, these variables did not correlate with the asthma control grade (data not shown).

**Table 1:** Mean + SD of the variables in asthmatic children and healthy controls

Variable	Asthmatic children	Healthy controls	*p*-value
TRP (μmol/L)	111.1 ± 41.0	67.4 ± 10.2	* *p* < < 0.01
Kyn (μmol/L)	2.68 ± 1.02	1.78 ± 0.42	* *p* < < 0.01
Kyn/TRP (μmol/mmol)	26.6 ± 18.5	26.7 ± 6.2	n.s.
Neopterin (nmol/L)	7.0 ± 3.8	5.9 ± 1.6	*p* = 0.0015

## Discussion

These findings confirm the complex network of cytokines involved in the asthma pathogenesis. Actually, the inflammatory cascade in asthma includes several actors: type 1, type 2, and type 3 cytokines, as recently evidenced [14, 15]. Therefore, different biomarkers, surrogate for different pathogenic pathways, may be detectable at the same time. In this regard, the current findings show that asthmatic children, recruited in a clinical practice setting, had increased both IDO pathway metabolites and neopterin, such as type 1 biomarker, together. On the other hand, both biomarker clusters did not discriminate the asthma control grade. This phenomenon might mean that increase of IDO pathway metabolites and neopterin secretion may be a signature of the underlying unbalanced immune regulation typical of allergic disorders.

In addition, there is a clear-cut association between tryptophan metabolism and the oxidative stress as pointed out by several studies [16–20].

This study has some limitation: cross-sectional design and lack of direct bronchial inflammatory markers.

In conclusion, IDO pathway metabolites and neopterin production are increased in children with allergic asthma and could represent a potential marker that could suggest the presence of a dysregulated immune response.

## Data Availability

All data analysed during this study are included in this published article and its supplementary information files.

## Abbreviations

ELISA: Enzyme-linked immunosorbent assay
FeNO: Fractioned exhaled NO
HPLC: High-performance liquid chromatography
IDO: Indoleamine 2,3-dioxygenase
Kyn: Kynurenine
NO: Nitric oxide
SAR: Seasobal allergic rhinitis
Trp: Tryptophan

## References

[1] Nakagome K, Nagata M (2018). Involvement and possible role of eosinophils in asthma exacerbation. Front Immunol.

[2] Mansur AH, Srivastava S, Sahal A (2018). Disconnect of type 2 biomarkers in severe asthma; dominated by FeNO as a predictor of exacerbations and periostin as predictor of reduced lung function. Resp Med.

[3] Denlinger LC, Phillips BR, Ramratnam S, Ross K, Bhakta NR, Cardet JC (2017). Inflammatory and comorbid features of patients with severe asthma and frequent exacerbations. Am J Respir Crit Care Med.

[4] Nowroozilarki N, Öz HH, Schroth C, Hector A, Nürnberg B, Hartl D, Kolahian S (2018). Anti-inflammatory role of CD11b^+^Ly6G^+^ neutrophilic cells in allergic airway inflammation in mice. Immunol Lett.

[5] Gostner JM, Becker K, Kofler H, Strasser B, Fuchs D (2016). Tryptophan metabolism in allergic disorders. Int Arch Allergy Immunol.

[6] von Bubnoff BT (2012). The indoleamine 2,3-dioxygenase (IDO) pathway controls allergy. Allergy.

[7] Kositz C, Schroecksnadel K, Grander G, Schennach H, Kofler H, Fuchs D (2008). Serum tryptophan concentration in patients predicts outcome of specific immunotherapy with pollen extracts. Int Arch Allergy Immunol.

[8] Ciprandi G, Amici M, Tosca M, Fuchs D (2010). Tryptophan metabolism in allergic rhinitis: the effect of pollen allergen exposure. Hum Immunol.

[9] Ciprandi G, De Amici M, Tosca M, Fuchs D, Marseglia G (2011). Serotonin in allergic rhinitis: a role for behavioural symptoms. Iran J Allergy Asthma Immunol.

[10] Ciprandi G, Tosca M, Fuchs D (2011). Nitric oxide metabolites in allergic rhinitis: the effect of pollen allergen exposure. Allergol Immunopathol.

[11] Wagener AH, de Nijs SB, Lutter R, Sousa AR, Weersink EJM, Bel EH (2015). External validation of blood eosinophils, FeNO and serum periostin as surrogates for sputum eosinophils in asthma. Thorax.

[12] Hu Y, Chen Z, Jin L, Wang M, Liao W (2017). Decreased expression of indolamine 2,3-dioxygenase in childhood allergic asthma and its inverse correlation with fractional concentration of exhaled nitric oxide. Ann Allergy Asthma Immunol.

[13] Global Initiative for Asthma. GINA guidelines. Global strategy for Asthma Management and Prevention. 2011. Available at: http://www.ginasthma.org/. Last access on Dec 2018.

[14] Ricciardolo F, Sorbello V, Folino A, Gallo F, Massaglia GM, Favatà G (2017). Identification of IL-17F/frequent exacerbator endotype in asthma. J Allergy Clin Immunol.

[15] Nassenstein C, Krasteva-Christ G, Renz H (2018). New aspects of neuroinflammation and neuroimmune crosstalk in the airways. J Allergy Clin Immunol.

[16] Leonardi S, Pecoraro R, Filippelli M, Miraglia del Giudice M, Marseglia G, Salpietro C (2014). Allergic reactions to foods by inhalation in children. Allergy Asthma Proc.

[17] Wigner P, Czarny P, Galecki P, Su KP, Sliwinski T (2018). The molecular aspects of oxidative & nitrosative stress and the tryptophan catabolites pathway (TRYCATs) as potential causes of depression. Psychiatry Res.

[18] Xu Kang, Liu Hongnan, Bai Miaomiao, Gao Jing, Wu Xin, Yin Yulong (2017). Redox Properties of Tryptophan Metabolism and the Concept of Tryptophan Use in Pregnancy. International Journal of Molecular Sciences.

[19] Wang Q, Liu D, Song P, Zou MH (2015). Tryptophan-kynurenine pathway is dysregulated in inflammation, and immune activation. Front Biosci (Landmark Ed).

[20] Zhuravlev AV, Vetrovoy OV, Savvateeva-Popova EV (2018). Enzymatic and non-enzymatic pathways of kynurenines’ dimerization: the molecular factors for oxidative stress development. PLoS Comput Biol.

